# Draft Genome Sequence of a Polydroxyalkanoate-Synthesizing Bacterium, *Bacillus* sp. Strain PJC48, Isolated from Activated Sludge

**DOI:** 10.1128/genomeA.01751-16

**Published:** 2017-03-09

**Authors:** Jin-Jin Gu, Ying Zhou, Jian-Jiang Lu, Bang-Ce Ye

**Affiliations:** a School of Chemistry and Chemical Engineering, Shihezi University, Xinjiang, China; b State Key Laboratory of Bioreactor Engineering, East China University of Science & Technology, Shanghai, China

## Abstract

The genome sequence of a *Bacillus* strain is capable of synthesizing polyhydroxyalkanoates, and *Bacillus* sp. is considered a platform strain for the production of many biodegradable materials. Here, we present the sequence of the PJC48 strain genome, which is composed of three chromatin structures, an extracellular structure, and a cytoskeleton.

## GENOME ANNOUNCEMENT

*Bacillius* is a genus of Gram-positive bacteria isolated from activated sludge. The bacterial strain PJC48 has been previously reported for its special ability, such as suppressing damping off. *Bacillius cereus* UW85 can produce two antibiotics, zwittermicin A (1) and kanosamine (2), and can also protect tobacco seeds from infection (3). However, there are few studies describing its ability to synthesize polyhydroxyalkanoates (PHAs). We utilized glucose as the sole carbon source and researched the optimal conditions for the synthesis of polyhydroxyalkanoates. The bacterial cultures were grown under aerobic conditions at 30°C for 48 h.

In this study, cell growth can be seen significantly in a lysogeny broth medium. According to PCR amplification analysis of 16S rRNA gene sequences, which was isolated from sludge, the highest sequence identity (99%) was observed with the 16S rDNA gene of Bacillus genus. *Bacillus* sp. PJC48 is one of three closely associated organisms (*Bacillus thuringiensis* strain 262AG1, *Bacillus* sp. enrichment culture clone SYW10, and *Bacillus* sp. strain WR-15) with which the previous information about members of the genus *Bacillus* were subdivided in accordance with 16S rRNA gene sequences and biochemical features. The genus *Bacillus* has the characteristics of Gram-positive, strictly aerobic, and rod-shaped bacteria. It belongs to the important characteristics of bacteria that can produce spores with special resistance under adverse conditions.

After completing the genomic DNA extraction by 1% agarose gel electrophoresis, the genomic DNA was detected, and 430-bp paired-end libraries were generated using Covaris M220. Libraries were constructed using the TruSeq reagent and DNA sample prep kits, based on their protocols, and sequenced using the MiSeq reagent kit version 3 by the Majorbio Company. There were some low-quality data from the raw data, which, in order to make the subsequent assembly quality more accurate, were sheared before the subsequent assembly. First, for the genome assembly, multiple k-mer parameters were spliced for optimized sequence using SOAP*denovo* version 2.04 (http://soap.genomics.org.cn) (4) and obtained the best resulting assembly. Second, the assembly result was filled and corrected using GapCloser version 1.12. After the original data were sheared, the clean data of 3,452,985 bp and two reads were obtained, consisting of 298× coverage of the genome. The assembly result has 5,855,511 bp of bases in all scaffolds.

The draft genome sequence has a total length of 4.85 Mb, with a G+C content of 35.7%, and it is composed of 100 contigs. Scaffolding produced 77 large contigs, of which the largest length (0.72 Mb) accounted for 12% of the total assembly length. Genes were predicted using Glimmer version 3.02 (http://ccb.jhu.edu/software/glimmer/index.shtml) (5), and 6,319 protein-coding genes, 69 tRNA genes, and two rRNA operons were identified with tRNAscan-SE (6). The gene is 768 bp in average length and accounts for 82.9% of the genome.

### Accession number(s).

This whole-genome shotgun project has been deposited at DDBJ/ENA/GenBank under the accession no. MSBQ00000000. The version described in this paper is version MSBQ01000000.
